# A Tablet-Based App for Carpal Tunnel Syndrome Screening: Diagnostic Case-Control Study

**DOI:** 10.2196/14172

**Published:** 2019-09-13

**Authors:** Koji Fujita, Takuro Watanabe, Tomoyuki Kuroiwa, Toru Sasaki, Akimoto Nimura, Yuta Sugiura

**Affiliations:** 1 Department of Orthopedic and Spinal Surgery Graduate School of Medical and Dental Sciences Tokyo Medical and Dental University Tokyo Japan; 2 School of Science for Open and Environmental Systems Graduate School of Science and Technology Keio University Yokohama Japan; 3 Functional Joint Anatomy Graduate School of Medical and Dental Sciences Tokyo Medical and Dental University Tokyo Japan

**Keywords:** carpal tunnel syndrome, screening test, movement, thumb

## Abstract

**Background:**

Carpal tunnel syndrome (CTS), the most common neuropathy, is caused by a compression of the median nerve in the carpal tunnel and is related to aging. The initial symptom is numbness and pain of the median nerve distributed in the hand area, while thenar muscle atrophy occurs in advanced stages. This atrophy causes failure of thumb motion and results in clumsiness; even after surgery, thenar atrophy does not recover for an extended period. Medical examination and electrophysiological testing are useful to diagnose CTS; however, visits to the doctor tend to be delayed because patients neglect the symptom of numbness in the hand. To avoid thenar atrophy-related clumsiness, early detection of CTS is important.

**Objective:**

To establish a CTS screening system without medical examination, we have developed a tablet-based CTS detection system, focusing on movement of the thumb in CTS patients; we examined the accuracy of this screening system.

**Methods:**

A total of 22 female CTS patients, involving 29 hands, and 11 female non-CTS participants were recruited. The diagnosis of CTS was made by hand surgeons based on electrophysiological testing. We developed an iPad-based app that recorded the speed and timing of thumb movements while playing a short game. A support vector machine (SVM) learning algorithm was then used by comparing the thumb movements in each direction among CTS and non-CTS groups with leave-one-out cross-validation; with this, we conducted screening for CTS in real time.

**Results:**

The maximum speed of thumb movements between CTS and non-CTS groups in each direction did not show any statistically significant difference. The CTS group showed significantly slower average thumb movement speed in the 3 and 6 o’clock directions (*P*=.03 and *P*=.005, respectively). The CTS group also took a significantly longer time to reach the points in the 2, 3, 4, 5, 6, 8, 9, and 11 o’clock directions (*P*<.05). Cross-validation revealed that 27 of 29 CTS hands (93%) were classified as having CTS, while 2 of 29 CTS hands (7%) did not have CTS. CTS and non-CTS were classified with 93% sensitivity and 73% specificity.

**Conclusions:**

Our newly developed app could classify disturbance of thumb opposition movement and could be useful as a screening test for CTS patients. Outside of the clinic, this app might be able to detect middle-to-severe-stage CTS and prompt these patients to visit a hand surgery specialist; this may also lead to medical cost-savings.

## Introduction

Carpal tunnel syndrome (CTS) is a common condition that causes numbness, tingling, and pain in the hand, and is caused by a compression of the median nerve in the carpal tunnel, a narrow passageway on the palm side of the wrist [1]. CTS is the most common neuropathy and affects 5%-10% of women over the age of 40 years [2]. The initial symptom of CTS is numbness of the hand from the thumb to the ring finger; as the condition progresses, atrophy of the thenar muscle occurs [3]. Thenar muscle atrophy is strongly connected to failure of thumb motion [4], which causes problems in daily life, such as difficulty with picking up small items, fastening buttons, and opening bottles.

Patients tend to delay seeing a doctor until the numbness worsens; thus, in most cases, thenar atrophy has occurred by the first hospital visit [5]. Early-stage CTS, prior to thenar atrophy, can be treated conservatively by using a night splint, anti-inflammatory injection, or surgical intervention [1]; however, for advanced-stage CTS with thenar atrophy, carpal tunnel release surgery is the first choice [3,6,7]. Despite carpal tunnel release surgery for CTS with thenar atrophy, recovery of the atrophy takes longer than a year after surgery in most cases [8]. Therefore, performing surgery before thenar atrophy develops is key to avoid inconvenience in daily life activities.

To diagnose CTS, physical examination techniques are used, such as testing for the Tinel sign or performing the Phalen maneuver and the compression maneuver [1]; however, the sensitivity and specificity of these tests are not high [9,10]. Electrophysiological testing—analyzing the conduction velocity in the median nerve— reflects the condition and compression of the nerve itself accurately [11,12]. However, this test requires not only a skillful technician, but also a dedicated and expensive machine; thus, this test is not widely available [13].

With recent advances in technology, mobile phone or tablet devices can now be used as a diagnostic tool in several diseases [14,15] and can enhance patients’ access to medical care in the early stage of disease [16]. Here, we developed a tablet app for CTS screening, focusing on thumb movement, and examined the usefulness of this app as a screening tool for CTS.

## Methods

### Study Design and Participant Recruitment

This study was approved by the Institutional Review Board of Tokyo Medical and Dental University. Paper-based informed consent was provided by all participants.

We recruited 22 female patients with CTS prior to surgery (CTS group, 29 hands) and 11 healthy female volunteers (non-CTS group, 11 hands) between January 2017 and July 2018. Upon recruitment, we obtained information about patients’ chief complaints and histories of hand trauma. For all patients, we performed physical examinations and CTS induction maneuvers and obtained x-ray images of their hands. In the CTS group, patients were included if they had a primary diagnosis of CTS and planned to undergo carpal tunnel release surgery. The criteria for primary CTS diagnosis included numbness of fingers; CTS-specific physical findings, such as positive results on a compression test or the Tinel sign, as well as the Phalen test; and an abnormal value in nerve conduction velocity, measured by Neuropack X1 (Nihon Kohden), based on Bland’s classification [17]. The following patients were excluded: patients with a history of hand injury or surgery; recurrence after release surgery of carpal tunnel; positive imaging findings indicative of first carpometacarpal or thumb metacarpophalangeal osteoarthritis, which could affect thumb motion; suspicion of disease on cervical spine; or positive magnetic resonance imaging findings of a space-occupying lesion in carpal tunnel.

As the control (non-CTS) group, female volunteers were included if they had undergone total hip arthroplasty in our hospital. We excluded patients from the non-CTS group if they had a history of wrist, hand, or finger injury or surgery; finger numbness; thumb pain; positive physical findings of CTS; or positive imaging findings of osteoarthritis of the first carpometacarpal or thumb metacarpophalangeal (see Table 1).

**Table 1:** Characteristics of participants in the CTS^a^ and non-CTS groups.

Participant characteristics		Non-CTS group	CTS group
Number of participants, N		11	22
Age in years, median (IQR^b^)		67 (58-74)	69 (59-80)
Sex (female), n (%)		11 (100)	22 (100)
Number of hands, n		11	29
**Bland’s classification**			
	Grade 1	N/A^c^	1
	Grade 2	N/A	3
	Grade 3	N/A	4
	Grade 4	N/A	9
	Grade 5	N/A	7
	Grade 6	N/A	5

^a^CTS: carpal tunnel syndrome.

^b^IQR: interquartile range.

^c^N/A: not applicable.

### App Design

**Figure 1:**
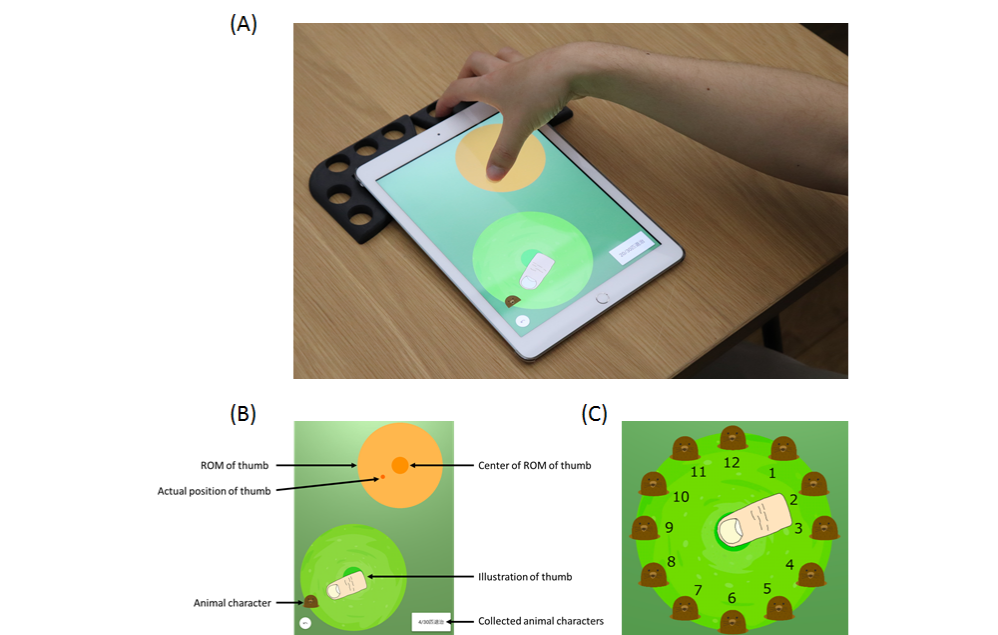
Participants used this app with the thumb of each hand. (A) The index to small fingers were fixed to the holder. When the patient touched the orange circle with their thumb, the illustration of the thumb appeared. (B) The patient collected animal characters by controlling the illustration. (C) Animal characters appeared in 12 clock-like directions centered on the green circle. ROM: range of motion.

The app was designed to run on a tablet device, in this case an iPad (Apple Inc); a 3D-printed holder, which fixed the fingers, was attached to the upper part of the tablet device in order to ensure use of only the thumb. A patient needed to slide the thumb along the touch screen to collect animal characters appearing on the screen (see Figure 1, A and B, and Multimedia Appendix 1). The animal characters appeared in 12 clock-like directions, in random order. In this way, it was possible to determine in which direction the patient’s thumb movement was restricted (see Figure 1, C). The animal characters completely disappeared after appearing twice from the ground. If the patient missed the animal character in one direction, a new animal character appeared. The animal characters appeared in 12 directions centering on a large green circle; the screening assessment was made based on the movement ability of the thumb centered on this circle. We designed the app such that when the animal character had been collected or completely disappeared, a new animal character appeared at the center of the circle and guided the patient to return the thumb to the center. Thus, the animal characters were set as markers to monitor thumb movement ability; whether the patient could collect the animal characters was not a factor in the screening test.

In this study, we used a two-class system to classify CTS or non-CTS using a support vector machine (SVM) [18]. SVM is a supervised learning algorithm for classification and regression analysis. In considering two-class classification, the boundary that distinguished between the two classes was called the decision boundary; the distance of the datum closest to the boundary for each class was called the margin. SVM determined the decision boundary to maximize the margin. The symptoms used as the basis for classification were comprehensively judged by the results of the electrophysiological testing and the medical examination by the hand surgeon. The training data were given by the maximum speed (cm/second), the average speed (cm/second), and the total time (seconds) of the thumb movement for the 12 directions in which the animal characters appeared. In total, 36 parameters per patient were obtained. We calculated the coincidence rate of the classification based on the electrophysiological testing and the app-based screening test.

### Statistical Analysis

**Figure 2:**
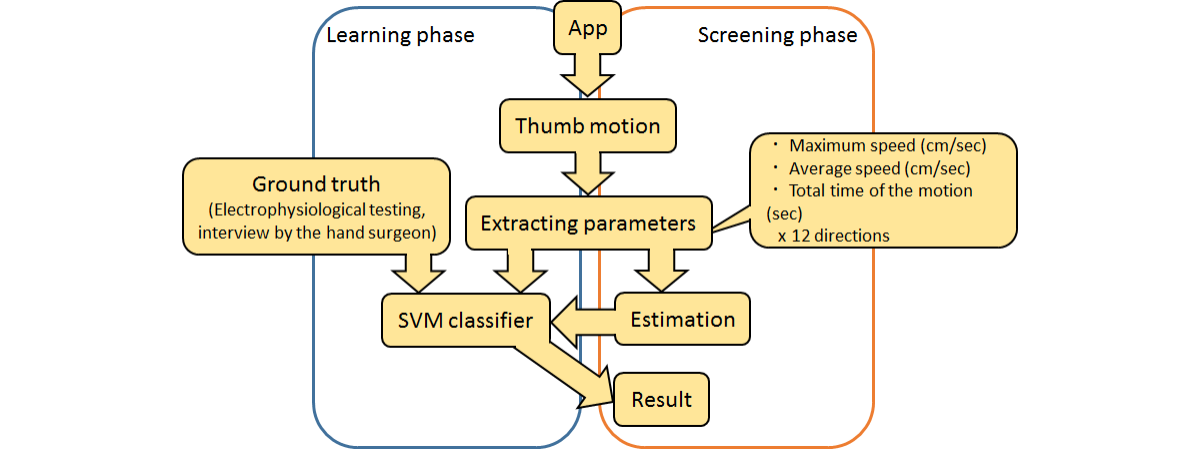
The flow of carpel tunnel syndrome (CTS) screening with the app. The app extracted three parameters from the thumb motion. The app monitored thumb movements in 12 directions centered on a circle; data on three parameters were collected for each of the 12 directions. The parameters were used for support vector machine (SVM) training in the learning phase and to classify CTS or non-CTS in the screening phase.

The raw data were collected in JavaScript Object Notation format and parsed to comma-separated value format. Hyperparameters used in SVM analyses were tuned using a grid search. The classification with the SVM adopted leave-one-out cross-validation. Leave-one-out cross-validation extracted one datum out of the dataset as a testing datum and used the rest as training data. This was repeated until each datum had been used as a testing datum. The SVM employed Python, version 3.7.0 (Python Software Foundation), and scikit-learn, version 0.20.2 (scikit-learn developers), a machine learning library (see Figure 2). The scikit-learn library played a role in the grid search, training, and validation. The Welch *t* test was used to compare changes between non-CTS and CTS individuals in each direction and was performed in R, version 3.5.1 (The R Foundation). A *P* value of less than .05 was considered as statistically significant.

## Results

**Figure 3:**
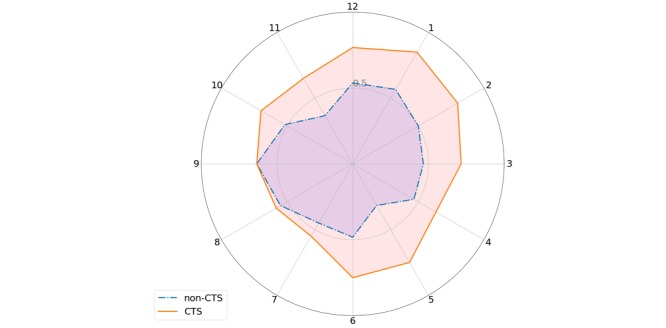
Representation of the median time to reach a point. The carpal tunnel syndrome (CTS) group took longer to reach most of the 12 points.

The maximum speed of thumb movements in the 12 directions was not significantly different (see Table 2). The CTS group showed significantly slower average thumb movement speed in the 3 and 6 o’clock directions (see Table 3) and also took significantly longer to reach the points in the 2, 3, 4, 5, 6, 8, 9, and 11 o’clock directions (see Table 4). Figure 3 shows the total thumb motion time.

**Figure 4:**
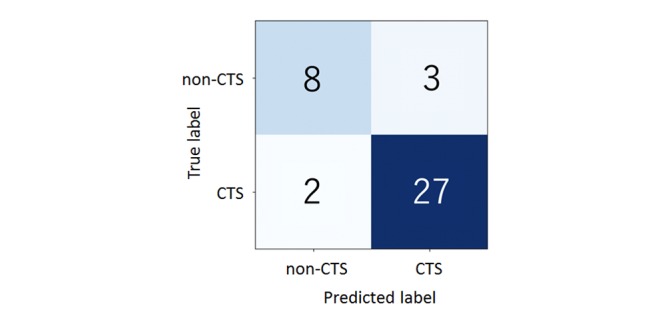
Representation of the median time in seconds to reach a point. The carpal tunnel syndrome (CTS) group took longer to reach most of the 12 points.

By leave-one-out cross-validation, 27 of 29 CTS hands (93%) were classified as having CTS, and 2 of 29 CTS hands (7%) were classified as not having CTS. CTS and non-CTS individuals were classified with 93% sensitivity and 73% specificity (see Figure 4).

**Table 2:** Radar chart data of the maximum speed of thumb movements in 12 directions.

Direction	Maximum speed of thumb movements (cm/second), median (95% CI)		*P* value
	Control (non-CTS^a^)	CTS	
12 o'clock	11.18 (7.79-21.89)	9.97 (8.56-14.52)	.56
1 o'clock	12.42 (9.45-20.22)	9.32 (7.73-12.11)	.11
2 o'clock	10.97 (7.30-17.37)	10.05 (8.37-13.65)	.94
3 o'clock	11.29 (8.35-15.64)	8.68 (7.45-13.19)	.34
4 o'clock	12.47 (9.21-16.00)	10.54 (8.89-14.99)	.48
5 o'clock	14.24 (8.98-22.14)	10.96 (8.71-14.19)	.06
6 o'clock	12.63 (6.82-16.12)	10.59 (7.94-12.73)	.47
7 o'clock	10.75 (6.07-19.30)	8.71 (6.40-12.62)	.40
8 o'clock	8.80 (7.39-16.78)	11.74 (6.90-15.52)	.58
9 o'clock	10.42 (6.47-15.53)	10.82 (6.03-12.85)	>.99
10 o'clock	10.70 (8.01-13.76)	9.50 (6.02-11.90)	.60
11 o'clock	10.81 (6.68-16.67)	9.18 (7.17-12.10)	.44

^a^CTS: carpal tunnel syndrome.

**Table 3:** Radar chart data of the average speed of thumb movements in 12 directions.

Direction	Average speed of thumb movements (cm/second), median (95% CI)		*P* value
	Control (non-CTS^a^)	CTS	
12 o'clock	7.22 (4.96-12.16)	5.20 (4.32-6.60)	.16
1 o'clock	7.75 (4.87-10.80)	5.57 (4.52-8.62)	.33
2 o'clock	6.14 (4.91-9.58)	4.77 (3.70-7.48)	.94
3 o'clock	7.48 (4.33-10.70)	5.59 (3.20-6.59)	.03
4 o'clock	6.98 (4.46-9.53)	6.10 (3.44-7.56)	.21
5 o'clock	13.48 (5.78-15.25)	6.71 (3.41-7.82)	.06
6 o'clock	7.53 (5.80-12.01)	4.90 (2.99-6.38)	.005
7 o'clock	6.82 (5.02-14.53)	5.41 (3.37-6.42)	.06
8 o'clock	6.67 (4.81-13.13)	4.95 (3.48-6.71)	.50
9 o'clock	7.18 (4.83-10.18)	6.06 (3.57-7.63)	.38
10 o'clock	7.64 (5.57-11.16)	4.76 (3.09-7.16)	.16
11 o'clock	8.54 (4.27-12.02)	4.32 (3.13-5.73)	.04

^a^CTS: carpal tunnel syndrome.

**Table 4:** Radar chart data of the total time of thumb movements in 12 directions.

Direction	Total time of thumb movements (seconds), median (95% CI)		*P* value
	Control (non-CTS^a^)	CTS	
12 o'clock	0.53 (0.33-0.90)	0.77 (0.58-0.97)	.11
1 o'clock	0.57 (0.38-0.93)	0.85 (0.63-1.10)	.34
2 o'clock	0.50 (0.40-0.88)	0.80 (0.68-1.10)	.01
3 o'clock	0.47 (0.37-1.00)	0.72 (0.55-1.30)	.01
4 o'clock	0.47 (0.38-0.58)	0.63 (0.57-1.07)	.01
5 o'clock	0.32 (0.23-0.50)	0.75 (0.50-1.18)	.003
6 o'clock	0.48 (0.35-0.58)	0.75 (0.60-1.40)	.01
7 o'clock	0.45 (0.27-0.65)	0.55 (0.45-0.95)	.13
8 o'clock	0.55 (0.25-0.80)	0.58 (0.40-1.08)	.02
9 o'clock	0.63 (0.40-0.73)	0.63 (0.58-0.92)	.02
10 o'clock	0.52 (0.32-0.75)	0.70 (0.43-0.95)	.06
11 o'clock	0.37 (0.30-0.82)	0.65 (0.43-1.12)	.01

^a^CTS: carpal tunnel syndrome.

## Discussion

### Principal Findings

In this study, we developed a screening app for tablet devices that could detect thumb movement. With this app, CTS was detected with 93% sensitivity, 73% specificity, and 90% positive predictive value, which is as good as a diagnosis based on maneuver tests by a doctor. Testing for the Tinel sign showed 62% sensitivity, 93% specificity, and 88% positive predictive value; the Phalen maneuver showed 96% sensitivity, 80% specificity, and 79% positive predictive value.

Some diseases show specific finger or hand movements (eg, tremor in Parkinson disease), and doctors typically focus on these movements during the medical examination to diagnose and monitor the disease [19]. However, such visual information is difficult to quantify. Some studies have attempted to quantify these movements by using sensors or cameras. Motion capture analysis of the finger in cervical spondylosis [20] and small three-axis gyroscope analysis of thumb movement in CTS [4] have been reported. These methods have advantages in allowing precise measurement of detailed motion; on the other hand, special and precise devices and techniques are needed to achieve this and they are difficult to apply in clinical practice or daily life.

Tremor and writing disturbance in Parkinson disease have been well studied, and tablet or watch devices have come into clinical use recently [21-23]. Obtaining electrocardiograms or blood pressure by touch sensor can help monitor health status [24,15]; in addition, eye or skin diseases can be diagnosed using mobile phone cameras, and mental status can be examined through speech tone using mobile phone apps [25]. Widely used tablets and mobile phones have touch sensors, gyroscopes, cameras, and global positioning system. Development of apps is also relatively easy; therefore, use of these devices to analyze and assess the status of diseases in the daily living environment has gained attention. Furthermore, compared to medical devices, tablets and mobile phones are much cheaper.

In this study, the correct classification rate of CTS was approximately 90%, which was comparable to that obtained with some diagnostic maneuvers specific to CTS. We focused on disturbance of thumb opposition movements and tracking the movements while playing a game. On the other hand, we misclassified approximately 10% of hands. This may be because thenar atrophy had proceeded for a couple of months or years; acute onset of CTS will not cause thenar atrophy at the time of this test. Also, we included the patients with mild CTS status without thenar atrophy or thumb movement disturbance. To improve the screening accuracy, we plan to include a detection method of sensory disturbance and subjective scoring of CTS into the screening algorithm. Subjective scoring has been well studied in the clinical field; the Boston Carpal Tunnel Syndrome Questionnaire or the Disabilities of the Arm, Shoulder, and Hand questionnaire are routinely used to assess patients’ status. These subjective questionnaires can be easily included into our app. With these developments, we plan to assess the severity of CTS using this app.

### Limitations

There are some limitations to this study. First, we did not analyze other diseases with finger numbness, such as cervical spondylosis, diabetes neuropathy, or cubital tunnel syndrome. Thus, the specificity of screening in this app has not been well addressed. Second, conditions associated with thumb movement, such as trapeziometacarpal joint arthritis or a history of hand fracture, were not assessed. However, compared to these conditions, CTS is much more common, and thus this app will be useful as a screening tool. Furthermore, we made a plastic holder for the index, middle, ring, and small fingers, but the design of this holder requires further consideration to ensure consistency.

### Conclusions

We showed that our newly developed app could classify disturbance of thumb opposition movement and can be useful as a screening test for CTS patients. Outside of the clinic, this app might be able to detect middle-to-severe-stage CTS and could prompt these patients to visit hand surgery specialists; this may also facilitate cost-savings.

## Appendix

Multimedia Appendix 1 The participant used this app with the thumb. The index to small fingers were fixed to the folder.

## Abbreviations

AIP-PRISM: Center for Advanced Intelligence Project—Public/Private R&D Investment Strategic Expansion PrograM
CTS: carpal tunnel syndrome
IQR: interquartile range
JST: Japan Science and Technology Agency
N/A: not applicable
PRESTO: Precursory Research for Embryonic Science and Technology
SVM: support vector machine

## References

[1] Wipperman J, Goerl K (2016). Carpal tunnel syndrome: Diagnosis and management. Am Fam Physician.

[2] Middleton SD, Anakwe RE (2014). Carpal tunnel syndrome. BMJ.

[3] Padua L, Coraci D, Erra C, Pazzaglia C, Paolasso I, Loreti C, Caliandro P, Hobson-Webb LD (2016). Carpal tunnel syndrome: Clinical features, diagnosis, and management. Lancet Neurol.

[4] Kuroiwa T, Fujita K, Nimura A, Miyamoto T, Sasaki T, Okawa A (2018). A new method of measuring the thumb pronation and palmar abduction angles during opposition movement using a three-axis gyroscope. J Orthop Surg Res.

[5] Fernandes CH, Meirelles LM, Raduan Neto J, Nakachima LR, Dos Santos JB, Faloppa F (2013). Carpal tunnel syndrome with thenar atrophy: Evaluation of the pinch and grip strength in patients undergoing surgical treatment. Hand (N Y).

[6] Carlson H, Colbert A, Frydl J, Arnall E, Elliot M, Carlson N (2010). Current options for nonsurgical management of carpal tunnel syndrome. Int J Clin Rheumtol.

[7] Kim P, Lee H, Kim T, Jeon I (2014). Current approaches for carpal tunnel syndrome. Clin Orthop Surg.

[8] Garg B, Manhas V, Vardhan A, Srivastava DN, Das CJ, Vibha D, Gupta V, Malhotra R, Kotwal P (2019). Thumb opposition recovery following surgery for severe carpal tunnel syndrome: A clinical, radiological, and electrophysiological pilot study. J Hand Surg Am.

[9] Kuhlman KA, Hennessey WJ (1997). Sensitivity and specificity of carpal tunnel syndrome signs. Am J Phys Med Rehabil.

[10] Wiesman IM, Novak CB, Mackinnon SE, Winograd JM (2003). Sensitivity and specificity of clinical testing for carpal tunnel syndrome. Can J Plast Surg.

[11] Sonoo M, Menkes DL, Bland JDP, Burke D (2018). Nerve conduction studies and EMG in carpal tunnel syndrome: Do they add value?. Clin Neurophysiol Pract.

[12] Werner RA, Andary M (2011). Electrodiagnostic evaluation of carpal tunnel syndrome. Muscle Nerve.

[13] Basiri K, Katirji B (2015). Practical approach to electrodiagnosis of the carpal tunnel syndrome: A review. Adv Biomed Res.

[14] Lipschitz J, Miller CJ, Hogan TP, Burdick KE, Lippin-Foster R, Simon SR, Burgess J (2019). Adoption of mobile apps for depression and anxiety: Cross-sectional survey study on patient interest and barriers to engagement. JMIR Ment Health.

[15] Jamaladin H, van de Belt TH, Luijpers LC, de Graaff FR, Bredie SJ, Roeleveld N, van Gelder MM (2018). Mobile apps for blood pressure monitoring: Systematic search in app stores and content analysis. JMIR Mhealth Uhealth.

[16] Uchino M, Kawashima M, Uchino Y, Suzuki N, Mitamura H, Mizuno M, Hori Y, Yokoi N, Tsubota K (2018). The evaluation of dry eye mobile apps for screening of dry eye disease and educational tear event in Japan. Ocul Surf.

[17] Bland JD (2000). A neurophysiological grading scale for carpal tunnel syndrome. Muscle Nerve.

[18] Noble WS (2006). What is a support vector machine?. Nat Biotechnol.

[19] Jankovic J (2008). Parkinson's disease: Clinical features and diagnosis. J Neurol Neurosurg Psychiatry.

[20] Omori M, Shibuya S, Nakajima T, Endoh T, Suzuki S, Irie S, Ariyasu R, Unenaka S, Sano H, Igarashi K, Ichimura S, Ohki Y (2018). Hand dexterity impairment in patients with cervical myelopathy: A new quantitative assessment using a natural prehension movement. Behav Neurol.

[21] Hubble RP, Naughton GA, Silburn PA, Cole MH (2015). Wearable sensor use for assessing standing balance and walking stability in people with Parkinson's disease: A systematic review. PLoS One.

[22] Rovini E, Maremmani C, Cavallo F (2017). How wearable sensors can support Parkinson's disease diagnosis and treatment: A systematic review. Front Neurosci.

[23] Wile DJ, Ranawaya R, Kiss ZH (2014). Smart watch accelerometry for analysis and diagnosis of tremor. J Neurosci Methods.

[24] Carpenter A, Frontera A (2016). Smart-watches: A potential challenger to the implantable loop recorder?. Europace.

[25] Faurholt-Jepsen M, Busk J, Frost M, Vinberg M, Christensen EM, Winther O, Bardram JE, Kessing LV (2016). Voice analysis as an objective state marker in bipolar disorder. Transl Psychiatry.

